# Supplementary material for: Aberrant regulation of the GSK‐3β/NRF2 axis unveils a novel therapy for adrenoleukodystrophy
Source: EMBO Mol Med. 2018 Jul 11;10(8):e8604. doi: 10.15252/emmm.201708604 (PMC6079538; doi:10.15252/emmm.201708604)
Supplement: Supplementary file 1 — Appendix [file EMMM-10-e8604-s001.pdf]

Appendix

Table of contents

Appendix Table S1. List of antibodies used in this study.

Appendix Table S2. List of Q-PCR probes used in this study.

Appendix Table S3. List of exact p values reported in this article.

Appendix Table S1. List of antibodies used in this study.

Antibody	Source	Reference	Dilution
8-oxo-dG	Abcam	62623	1:1000
AKT	Cell Signaling	4685	1:1000
Aldolase A	Novus Biologicals	600-915	1:5000
APP	AbD Serotec	AHP538	1:100
Cytochrome C	BD Biosciences Pharmigen	55643	1:500
GFAP	Dako	Z0334	1:500
GSK-3β	Cell Signaling	9315	1:1000
IBA1	Wako	019-19741	1:1000
Lamin B1	Abcam	16048	1:10000
Neurofilament H non-phophorylated (SMI32)	Covance Antibody-Biolegend	801701	1:3000
NRF2	Abcam	31163	1:500
pSer473 AKT	Cell Signaling	4060	1:1000
pSer9/21 GSK-3β	Cell Signaling	9331	1:1000
pThr308 AKT	Cell Signaling	13038	1:1000
pTyr216/279 GSK-3β	EMD Millipore	05-413	1:1000
Synaptophysin	Leica Biosystems	299-L-CE	1:500
γ-Tubulin	Sigma-Aldrich	T6557	1:20000
**Antibody (secondary)**	**Source**	**Reference**	**Dilution**
Goat anti-rabbit HRP	Dako	P0448	1:10000
Goat anti-mouse HRP	Dako	P0447	1:15000
Rabbit anti-goat HRP	Dako	P0449	1:15000

Appendix Table S2. List of Q-PCR probes used in this study.

Gene	Taqman ref.
Cebpα	Mm00514283
Ccl5	Mm01302427
Ccr6	Mm99999114
Chil3	Mm00657889
Cxcl9	Mm00434946
Cxcl10	Mm00445235
Cxcl12	Mm00457276
Gstα3	Mm01233706
Hmox1	Mm00516005
Igf1	Mm00439560
Il10	Mm00439614
Il1β	Mm01336189
Il6	Mm00446190
Mif	Mm01611157
Nfκb2	Mm00479807
Nqo1	Mm01253561
Nrf1	Mm00447996
Pgc-1α	Mm00447183
Retnla	Mm00445109
Rplp0	Mm01974474
Sirt1	Mm00490758
Tfam	Mm00447485
Tgfβ1	Mm01178820
Tnfα	Mm00443258
Tnfrsf1a	Mm01182929
GCLC	Hs00155249
HMOX1	Hs01110250
NQO1	Hs00168547
RPLP0	Hs99999902

Appendix Table S3. List of exact p values reported in this article

Figures	Parameter	Comparisons	P value
Figure 1A	NRF2	WT vs *Abcd1*⁻	0.031
Figure 1B	*Hmox1*	WT vs *Abcd1*⁻	0.022
Figure 1B	*Nqo1*	WT vs *Abcd1*⁻	0.028
Figure 1B	*Gstα3*	WT vs *Abcd1*⁻	0.033
Figure 1D	pThr308 AKT	WT vs *Abcd1*⁻	0.020
Figure 1D	pSer9 GSK-3β	WT vs *Abcd1*⁻	0.014
Figure 2B	Nucleus NRF2	CTL veh vs CTL + C26:0	0.043
Figure 2B	Nucleus NRF2	CTL veh vs CTL + oligomycin	<0.0001
Figure 2B	Cytoplasm NRF2	CTL veh vs CTL + C26:0	0.034
Figure 2C	*HMOX1*	CTL veh vs CTL + C26:0	0.002
Figure 2C	*HMOX1*	CTL veh vs CTL + oligomycin	0.011
Figure 2C	*NQO1*	CTL veh vs CTL + C26:0	0.004
Figure 2C	*NQO1*	CTL veh vs CTL + oligomycin	0.002
Figure 2C	*GCLC*	CTL veh vs CTL + C26:0	<0.0001
Figure 2C	*GCLC*	CTL veh vs CTL + oligomycin	0.029
Figure 2E	pSer473 AKT	CTL veh vs CTL + C26:0	0.046
Figure 2E	pSer473 AKT	CTL veh vs CTL + oligomycin	0.034
Figure 2E	pThr308 AKT	CTL veh vs CTL + C26:0	0.029
Figure 2E	pThr308 AKT	CTL veh vs CTL + oligomycin	0.008
Figure 2E	pSer9 GSK-3β	CTL veh vs CTL + C26:0	0.002
Figure 2E	pSer9 GSK-3β	CTL veh vs CTL + oligomycin	0.036
Figure 2F	*HMOX1*	CTL veh vs CTL + C26:0	0.003
Figure 2F	*HMOX1*	CTL + C26:0 vs CTL + C26:0 + SB216763	0.008
Figure 2F	*HMOX1*	CTL + C26:0 + SB216763 vs CTL + SB216763	0.025
Figure 2F	*NQO1*	CTL veh vs CTL + C26:0	0.006
Figure 2F	*GCLC*	CTL veh vs CTL + C26:0	0.0007
Figure 2F	*GCLC*	CTL + C26:0 vs CTL + C26:0 + C99021	0.007
Figure 2F	*GCLC*	CTL + C26:0 vs CTL + C26:0 + SB216763	0.0009
Figure 2F	*GCLC*	CTL veh vs CTL + SB216763	0.029
Figure 2F	*HMOX1*	X-ALD + C26:0 vs X-ALD + C26:0 + C99021	0.0003
Figure 2F	*HMOX1*	X-ALD + C26:0 vs X-ALD + C26:0 + SB216763	<0.0001
Figure 2F	*HMOX1*	X-ALD + C26:0 + C99021 vs X-ALD + C99021	0.028
Figure 2F	*HMOX1*	X-ALD + C26:0 + SB216763 vs X-ALD + SB216763	0.0008
Figure 2F	*NQO1*	X-ALD + C26:0 vs X-ALD + C26:0 + C99021	0.0007
Figure 2F	*NQO1*	X-ALD + C26:0 vs X-ALD + C26:0 + SB216763	0.0007
Figure 2F	*NQO1*	X-ALD + C26:0 + SB216763 vs X-ALD + SB216763	0.038
Figure 2F	*GCLC*	X-ALD + C26:0 vs X-ALD + C26:0 + C99021	0.02
Figure 2F	*GCLC*	X-ALD + C26:0 vs X-ALD + C26:0 + SB216763	0.002
Figure 3A	NRF2	WT vs *Abcd1*⁻	0.043
Figure 3A	NRF2	*Abcd1*⁻ vs *Abcd1*⁻ + DMF	0.031
Figure 3B	*Hmox1*	WT vs *Abcd1*⁻	0.041
Figure 3B	*Hmox1*	*Abcd1*⁻ vs *Abcd1*⁻ + DMF	0.032
Figure 3B	*Nqo1*	WT vs *Abcd1*⁻	0.0005
Figure 3B	*Nqo1*	*Abcd1*⁻ vs *Abcd1*⁻ + DMF	0.013
Figure 3B	*Gstα3*	WT vs *Abcd1*⁻	0.009

Figure 3B	*Gst*α*3*	*Abcd1*$^{-}$ vs *Abcd1*$^{-}$ + DMF	0.041
Figure 3C	AASA	WT vs *Abcd1*$^{-}$	0.048
Figure 3C	AASA	*Abcd1*$^{-}$ vs *Abcd1*$^{-}$ + DMF	0.011
Figure 3C	CEL	WT vs *Abcd1*$^{-}$	0.009
Figure 3C	CEL	*Abcd1*$^{-}$ vs *Abcd1*$^{-}$ + DMF	0.002
Figure 3C	CML	WT vs *Abcd1*$^{-}$	0.0097
Figure 3C	CML	*Abcd1*$^{-}$ vs *Abcd1*$^{-}$ + DMF	0.0014
Figure 3C	MDAL	WT vs *Abcd1*$^{-}$	0.0013
Figure 3C	MDAL	*Abcd1*$^{-}$ vs *Abcd1*$^{-}$ + DMF	0.002
Figure 3D	mtDNA	WT vs *Abcd1*$^{-}$	<0.0001
Figure 3D	mtDNA	*Abcd1*$^{-}$ vs *Abcd1*$^{-}$ + DMF	0.011
Figure 3E	*Sirt1*	WT vs *Abcd1*$^{-}$	0.005
Figure 3E	*Sirt1*	*Abcd1*$^{-}$ vs *Abcd1*$^{-}$ + DMF	0.015
Figure 3E	*Pgc-1*α	WT vs *Abcd1*$^{-}$	0.037
Figure 3E	*Pgc-1*α	*Abcd1*$^{-}$ vs *Abcd1*$^{-}$ + DMF	0.015
Figure 3E	*Nrf1*	WT vs *Abcd1*$^{-}$	<0.0001
Figure 3E	*Nrf1*	*Abcd1*$^{-}$ vs *Abcd1*$^{-}$ + DMF	0.007
Figure 3E	*Tfam*	WT vs *Abcd1*$^{-}$	0.001
Figure 3E	*Tfam*	*Abcd1*$^{-}$ vs *Abcd1*$^{-}$ + DMF	0.012
Figure 3F	ATP	WT vs *Abcd1*$^{-}$	0.013
Figure 3F	ATP	*Abcd1*$^{-}$ vs *Abcd1*$^{-}$ + DMF	0.023
Figure 4A	*Nf*κ*b2*	WT vs *Abcd1*$^{-}$	<0.0001
Figure 4A	*Nf*κ*b2*	*Abcd1*$^{-}$ vs *Abcd1*$^{-}$ + DMF	0.004
Figure 4A	*Il1*β	WT vs *Abcd1*$^{-}$	0.008
Figure 4A	*Il1*β	*Abcd1*$^{-}$ vs *Abcd1*$^{-}$ + DMF	0.007
Figure 4A	*Il6*	WT vs *Abcd1*$^{-}$	0.028
Figure 4A	*Il6*	*Abcd1*$^{-}$ vs *Abcd1*$^{-}$ + DMF	0.022
Figure 4A	*Tnf*α	WT vs *Abcd1*$^{-}$	0.031
Figure 4A	*Tnf*α	*Abcd1*$^{-}$ vs *Abcd1*$^{-}$ + DMF	0.035
Figure 4A	*Tnfrs1a*	WT vs *Abcd1*$^{-}$	0.016
Figure 4A	*Ccl5*	WT vs *Abcd1*$^{-}$	0.010
Figure 4A	*Ccl5*	*Abcd1*$^{-}$ vs *Abcd1*$^{-}$ + DMF	0.049
Figure 4A	*Cxcl9*	WT vs *Abcd1*$^{-}$	0.0001
Figure 4A	*Cxcl10*	WT vs *Abcd1*$^{-}$	0.005
Figure 4A	*Cxcl10*	*Abcd1*$^{-}$ vs *Abcd1*$^{-}$ + DMF	0.028
Figure 4A	*Ccr6*	WT vs *Abcd1*$^{-}$	0.013
Figure 4A	*Ccr6*	*Abcd1*$^{-}$ vs *Abcd1*$^{-}$ + DMF	0.047
Figure 4B	*Chil3*	WT vs *Abcd1*$^{-}$	0.033
Figure 4B	*Chil3*	*Abcd1*$^{-}$ vs *Abcd1*$^{-}$ + DMF	0.009
Figure 4B	*Fizz1*	WT vs *Abcd1*$^{-}$	0.006
Figure 4B	*Cxcl12*	WT vs *Abcd1*$^{-}$	0.0006
Figure 4B	*Cxcl12*	*Abcd1*$^{-}$ vs *Abcd1*$^{-}$ + DMF	0.043
Figure 4B	*Il10*	*Abcd1*$^{-}$ vs *Abcd1*$^{-}$ + DMF	0.0005
Figure 4B	*Igf1*	WT vs *Abcd1*$^{-}$	0.0011
Figure 4B	*Igf1*	*Abcd1*$^{-}$ vs *Abcd1*$^{-}$ + DMF	0.046
Figure 4B	*Mif*	WT vs *Abcd1*$^{-}$	<0.0001
Figure 4B	*Mif*	*Abcd1*$^{-}$ vs *Abcd1*$^{-}$ + DMF	0.005
Figure 4B	*Tgf*β*1*	WT vs *Abcd1*$^{-}$	0.0006
Figure 4B	*Tgf*β*1*	*Abcd1*$^{-}$ vs *Abcd1*$^{-}$ + DMF	0.030
Figure 5Y	Iba1^{+} cells/mm^{2}	WT vs DKO	0.0004
Figure 5Y	Iba1^{+} cells/mm^{2}	DKO vs DKO + DMF	0.003
Figure 5Y	GFAP^{+} cells/mm^{2}	WT vs DKO	0.003
Figure 5Y	GFAP^{+} cells/mm^{2}	DKO vs DKO + DMF	0.004
Figure 5Y	Synaptophysin	WT vs DKO	<0.001
Figure 5Y	Synaptophysin	DKO vs DKO + DMF	<0.001
Figure 5Y	APP	WT vs DKO	0.0082

Figure 5Y	APP	DKO vs DKO + DMF	0.019
Figure 5Z	Number of slips	WT vs DKO	< 0.0001
Figure 5Z	Number of slips	DKO vs DKO + DMF	0.036
Figure 5Z	Time to cross (s)	WT vs DKO	0.003
Figure 5Z	Time to cross (s)	DKO vs DKO + DMF	0.027
Figure 5Z	Number of shocks	WT vs DKO	0.004
Figure 5Z	Number of shocks	DKO vs DKO + DMF	0.006
Figure 5Z	Time of shocks (s)	WT vs DKO	0.013
Figure 5Z	Time of shocks (s)	DKO vs DKO + DMF	0.013
Figure EV2	C26:0	WT vs *Abcd1*$^{-}$	< 0.001
Figure EV2	C26:0	*Abcd1*$^{-}$ vs *Abcd1*$^{-}$ + DMF	0.037
Figure EV2	C24:0/C22:0	WT vs *Abcd1*$^{-}$	0.0016
Figure EV2	C26:0/C22:0	WT vs *Abcd1*$^{-}$	< 0.001
Figure EV3	*HMOX1*	CTL veh vs CTL C26:0	0.003
Figure EV3	*HMOX1*	CTL veh vs CTL DMF	0.006
Figure EV3	*NQO1*	CTL veh vs CTL C26:0	0.005
Figure EV3	*NQO1*	CTL veh vs CTL DMF	0.025
Figure EV3	*GCLC*	CTL veh vs CTL C26:0	0.0007
Figure EV3	*GCLC*	CTL C26:0 vs CTL C26:0 + DMF	0.016
Figure EV3	*HMOX1*	X- ALD C26:0 vs X-ALD C26:0 + DMF	<0.0001
Figure EV3	*HMOX1*	X-ALD veh vs X-ALD DMF	<0.0001
Figure EV3	*NQO1*	X- ALD C26:0 vs X-ALD C26:0 + DMF	<0.0001
Figure EV3	*NQO1*	X- ALD C26:0 + DMF vs X-ALD DMF	0.031
Figure EV3	*GCLC*	X- ALD C26:0 vs X-ALD C26:0 + DMF	0.0004
Figure EV3	*GCLC*	X- ALD C26:0 + DMF vs X-ALD DMF	0.018
