# Supplementary material for: Aberrant regulation of the GSK‐3β/NRF2 axis unveils a novel therapy for adrenoleukodystrophy
Source: EMBO Mol Med. 2018 Jul 11;10(8):e8604. doi: 10.15252/emmm.201708604 (PMC6079538; doi:10.15252/emmm.201708604)
Supplement: Supplementary file 2 — Expanded View Figures PDF [file EMMM-10-e8604-s002.pdf]

Expanded View Figures

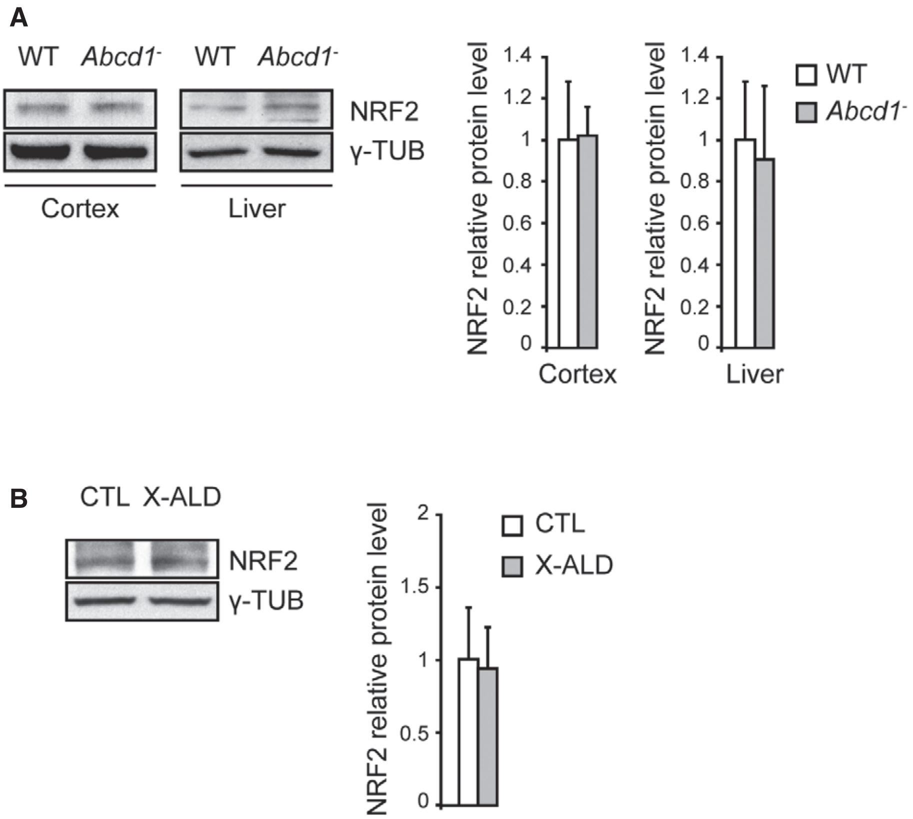

Figure EV1. Basal NRF2 levels in other mice tissues and human fibroblasts.

A Representative immunoblots of NRF2 protein level in WT (n = 8) and *Abcd1*$^{-}$ (n = 8) mice cerebral cortex and liver at 12 months of age. Protein levels normalized relative to γ-tubulin (γ-TUB) and quantification depicted as fold change to WT mice.

B Representative immunoblots of NRF2 protein level in total extracts from CTL (n = 5) and X-ALD (n = 5) fibroblasts. Protein levels normalized relative to γ-TUB. Quantification is depicted as fold change to control fibroblasts.

Data information: Data are presented as mean ± SD.

© 2018 The Authors

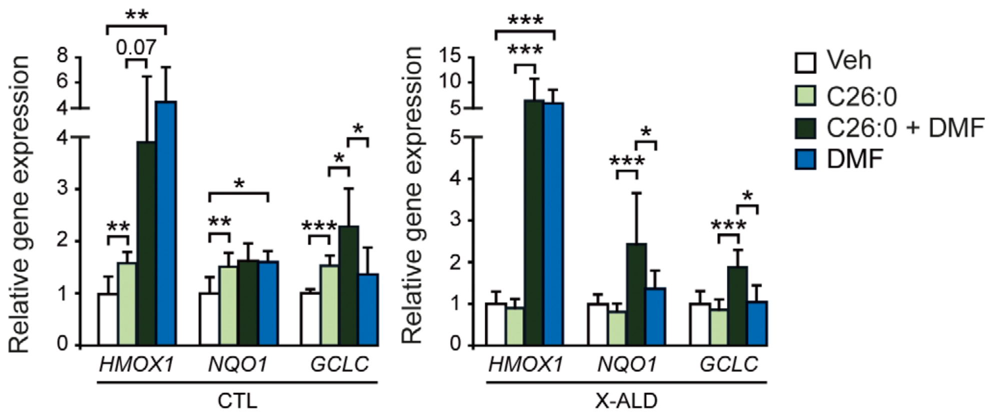

Figure EV2. DMF effect on NRF2 response in X-ALD fibroblasts.

Expression of NRF2 target genes was measured in CTL (n = 3) and X-ALD (n = 3) after C26:0 (50 μM, 24 h) and/or DMF (20 μM, 6 h). Gene expression normalized relative to *RPLP0*. Quantification depicted as fold change to vehicle-treated (Veh) fibroblasts. Data are presented as mean ± SD. *P < 0.05, **P < 0.01, ***P < 0.001 (one-way ANOVA followed by Tukey's *post hoc* test). See the exact P-values in Appendix Table S3.

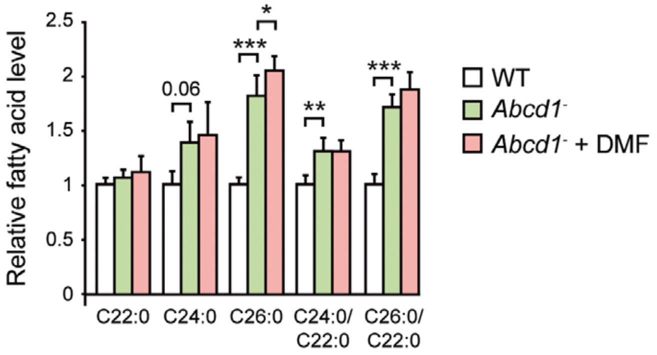

Figure EV3. VLCFA levels after DMF treatment in the spinal cord of X-ALD mouse model.

Saturated fatty acids 22:0, 24:0, and 26:0 level in the spinal cord of 12-month-old WT, *Abcd1*⁻, and *Abcd1*⁻ + DMF mice (n = 5). Fatty acid quantification was done by gas–liquid chromatography (see Materials and Methods). VLCFA 24:0 and 26:0 levels are normalized to the long-chain fatty acid 22:0 in the 26:0/22:0 and the 24:0/22:0 ratios. Quantification is depicted as fold change to WT mice. Data are presented as mean ± SD. *P < 0.05, **P < 0.01, ***P < 0.001 (one-way ANOVA followed by Tukey's *post hoc* test). See the exact P-values in Appendix Table S3.

© 2018 The Authors
