# Supplementary figures and images for: Aberrant regulation of the GSK‐3β/NRF2 axis unveils a novel therapy for adrenoleukodystrophy
Source: EMBO Mol Med. 2018 Jul 11;10(8):e8604. doi: 10.15252/emmm.201708604 (PMC6079538; doi:10.15252/emmm.201708604)

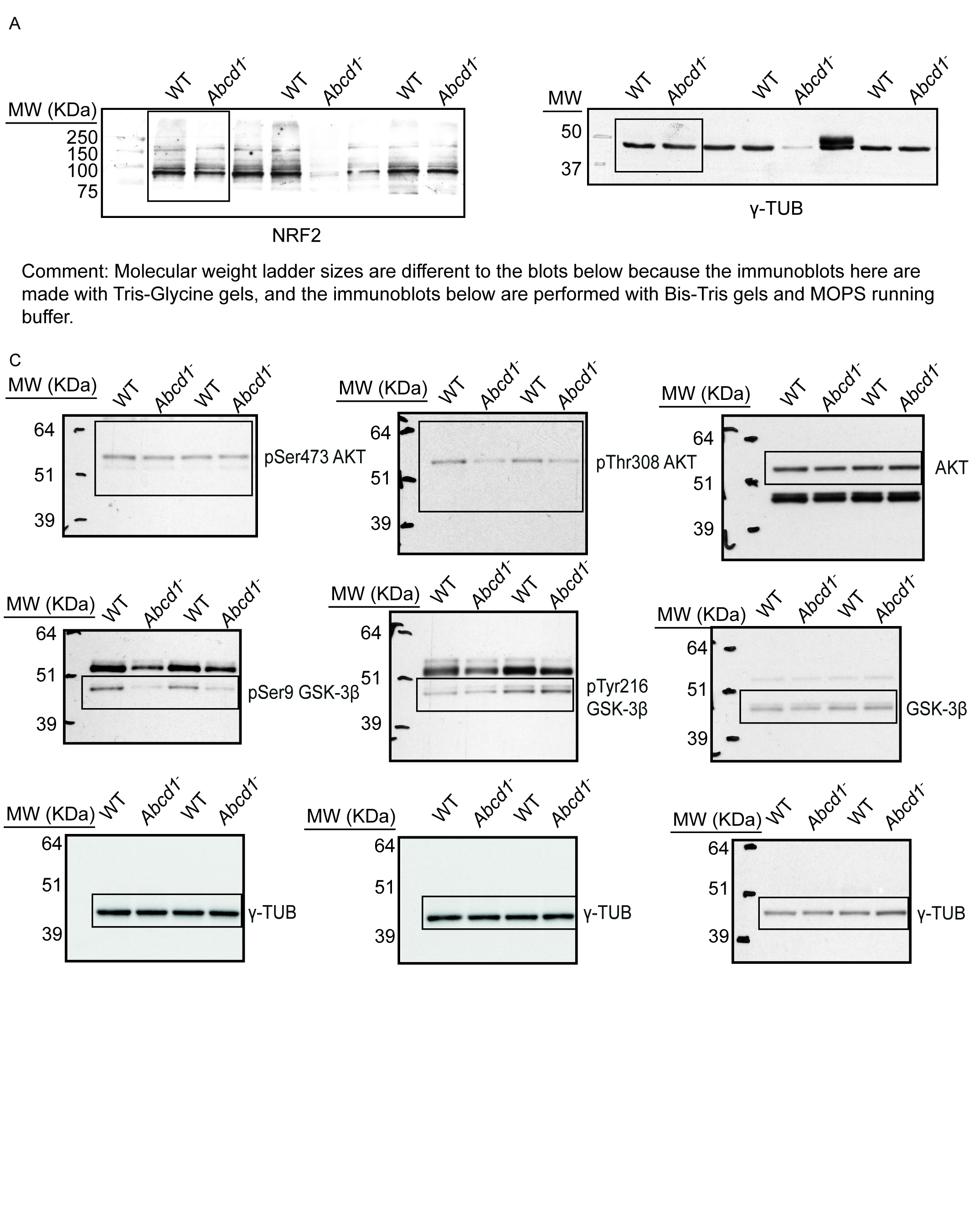

Supplement: Supplementary file 4 — Source Data for Figure 1 [file EMMM-10-e8604-s003.tif]

Source Data for Figure 2A

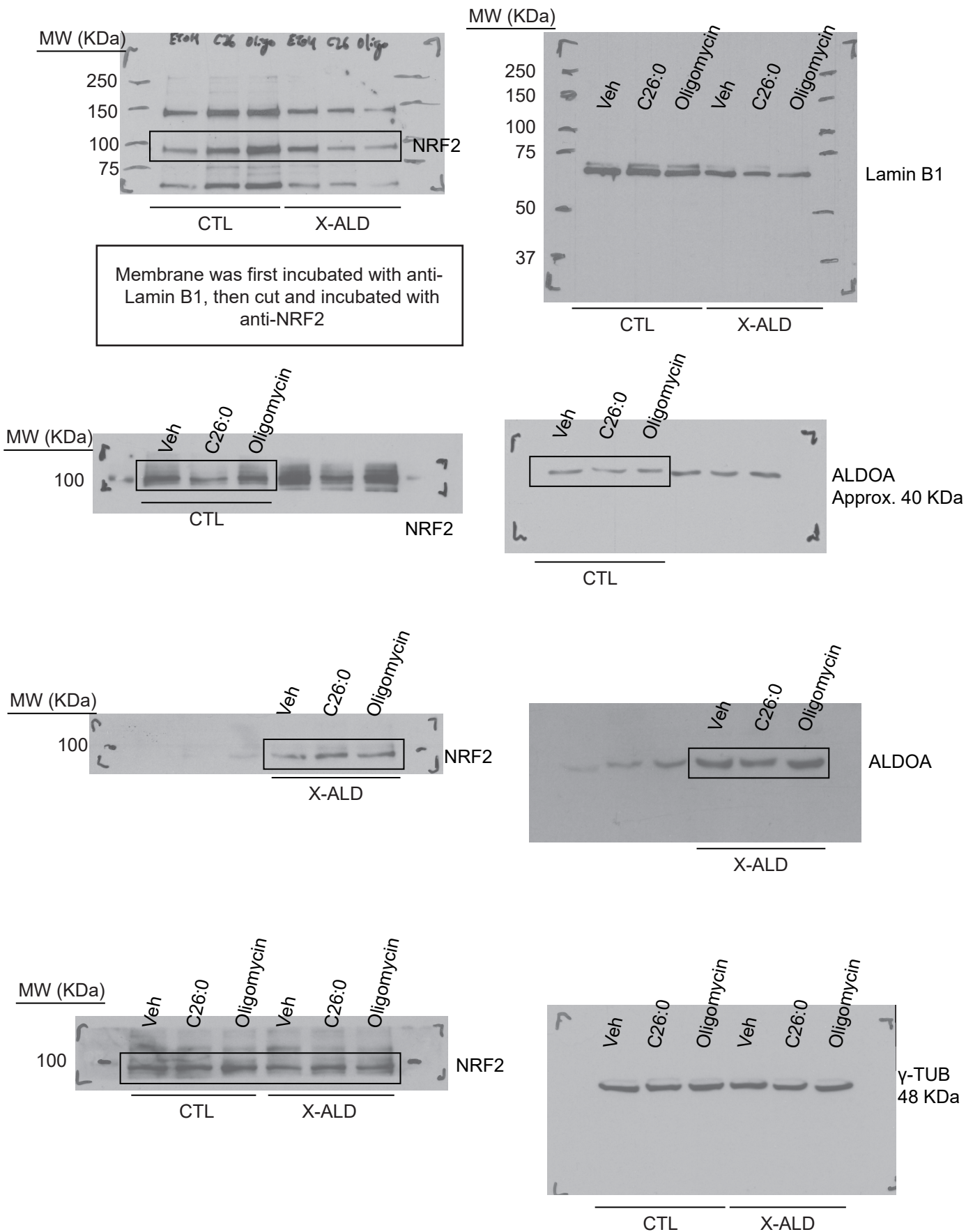

Source Data for Figure 2D

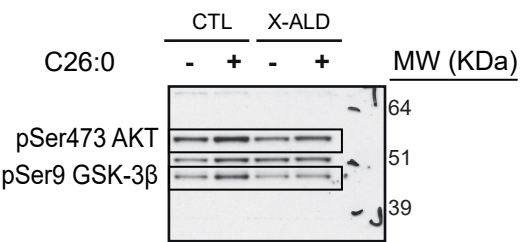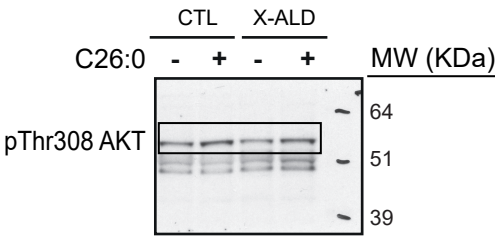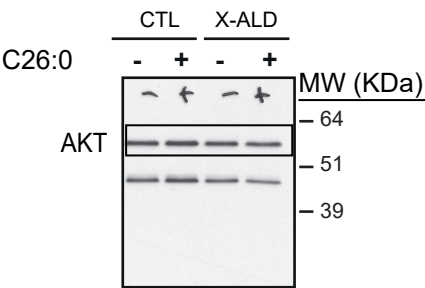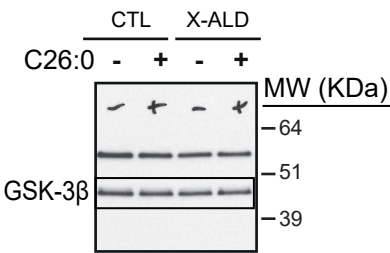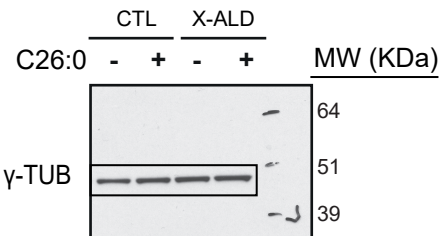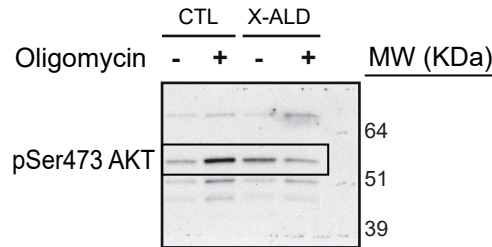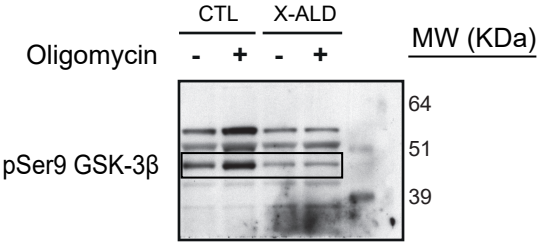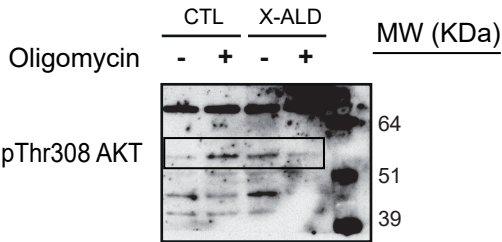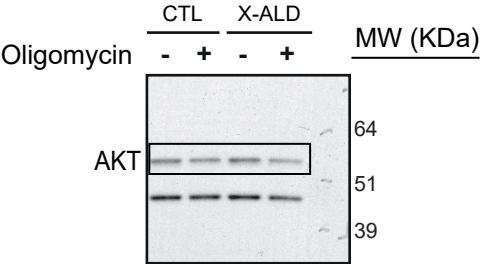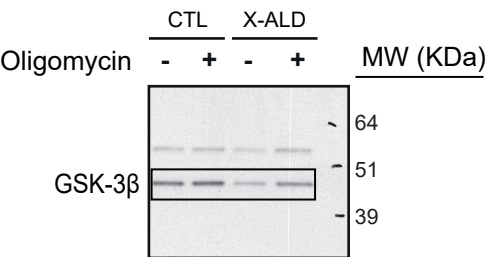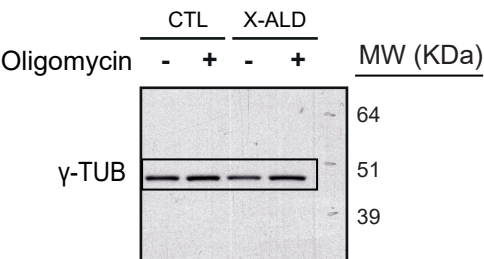

Supplement: Supplementary file 5 — Source Data for Figure 2 [file EMMM-10-e8604-s004.pdf]

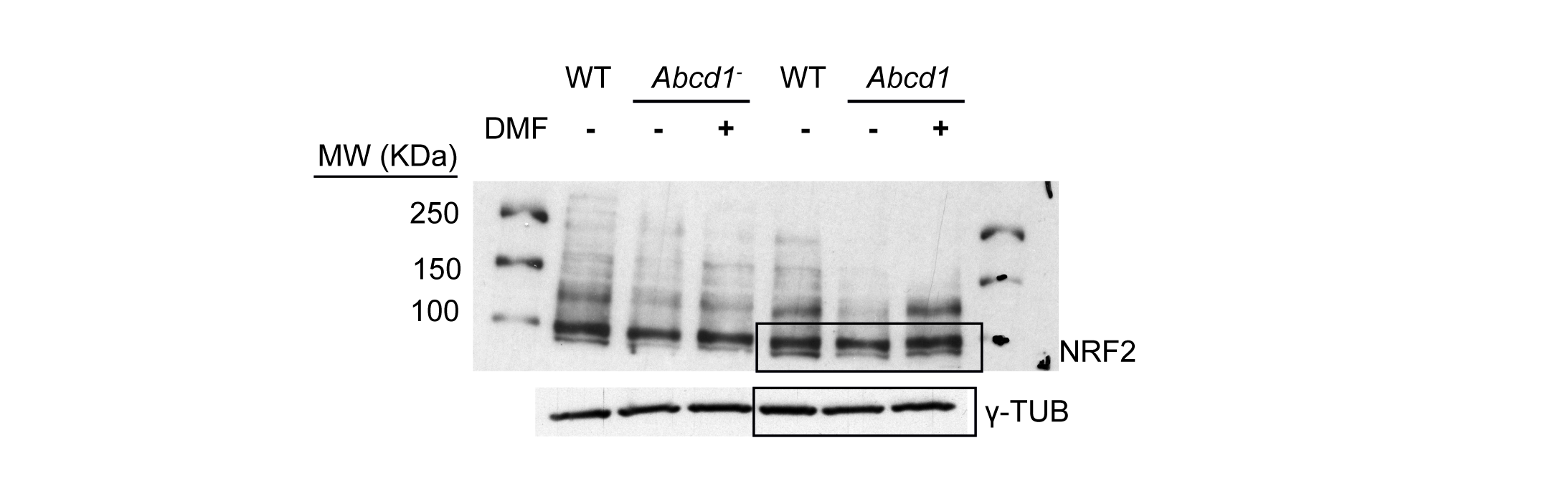

Supplement: Supplementary file 6 — Source Data for Figure 3 [file EMMM-10-e8604-s005.tif]
